# Dual Role of Metallic Trace Elements in Stress Biology—From Negative to Beneficial Impact on Plants

**DOI:** 10.3390/ijms20133117

**Published:** 2019-06-26

**Authors:** Ewa Muszyńska, Mateusz Labudda

**Affiliations:** 1 Department of Botany, Faculty of Agriculture and Biology, Warsaw University of Life Sciences-SGGW, Nowoursynowska 159, Building 37, 02-776 Warsaw, Poland; 2 Department of Biochemistry, Faculty of Agriculture and Biology, Warsaw University of Life Sciences-SGGW, Nowoursynowska 159, Building 37, 02-776 Warsaw, Poland

**Keywords:** hormesis, metal-induced enzymes activity, metallophyte, toxicity, trace elements

## Abstract

Heavy metals are an interesting group of trace elements (TEs). Some of them are minutely required for normal plant growth and development, while others have unknown biological actions. They may cause injury when they are applied in an elevated concentration, regardless of the importance for the plant functioning. On the other hand, their application may help to alleviate various abiotic stresses. In this review, both the deleterious and beneficial effects of metallic trace elements from their uptake by roots and leaves, through toxicity, up to the regulation of physiological and molecular mechanisms that are associated with plant protection against stress conditions have been briefly discussed. We have highlighted the involvement of metallic ions in mitigating oxidative stress by the activation of various antioxidant enzymes and emphasized the phenomenon of low-dose stimulation that is caused by non-essential, potentially poisonous elements called hormesis, which is recently one of the most studied issues. Finally, we have described the evolutionary consequences of long-term exposure to metallic elements, resulting in the development of unique assemblages of vegetation, classified as metallophytes, which constitute excellent model systems for research on metal accumulation and tolerance. Taken together, the paper can provide a novel insight into the toxicity concept, since both dose- and genotype-dependent response to the presence of metallic trace elements has been comprehensively explained.

## 1. Introduction

Mineral nutrients are the chemical elements that are required by plants to perform vital metabolic activities. Therefore, researchers have been interested in the identification of organism needs for essential elements for many years in order to avoid nutrient deficiency as well as increase the size and quality of crop yield [1,2]. Currently, special attention is paid to necessary elements that may bring about opposite effects and induce severe disturbances when they are applied in elevated concentrations [3]. Besides biological functions, the elements can be also divided on the basis of their relative concentration in organs. Trace elements (TEs), whose concentration in plant tissues is very low (ppb range or less than 10 ppm), should be mentioned in this quantity-based classification [4]. Among TEs, there is a group of ions that are commonly known as heavy metals (HMs), which belong to the most dangerous chemicals in ecotoxicology.

The presence of TEs in the environment may be caused by either natural or by anthropogenic activities [5,6,7]. The natural TEs sources do not lead to environmental contamination, while the latter is the major reason of metal accumulation in the surrounding world, and therefore may increase the risk of contaminants spreading over long distances by surface and ground waters, wind, and/or herbivores [8,9]. Consequently, HMs may contribute a serious threat to different levels of the food chain, since plants are the first link in this linear network. Although some HMs, such as Co (cobalt), Cu (copper), Fe (iron), Mn (manganese), Ni (nickel), Se (selenium), and Zn (zinc), are essential or beneficial for plant growth and development, they can easily lead to poisoning when their concentration rises to supra-optimal values. On the contrary, other elements, such as As (arsenic), Cd (cadmium), Cr (chromium), Hg (mercury), and Pb (lead), are not required from the physiological point of view and they may evoke toxicity at a much lower concentration than the first group. What is more, a number of TEs are dispersed in the environment for a long time. In the soils of temperate climate, the half-life time of chosen TEs varies from 75 to 380 years for Cd and from 1000 to 3000 years for Cu, Ni, Pb, Zn, and Se [4]. For this reason, all of these elements are considered to be non-biodegradable and persistent; however, they can be partially removed from the place where they are accumulated by the natural ability of plant species to uptake HMs via root or foliar penetration [7,10,11].

In spite of the fact that any authoritative body, such as the International Union of Pure and Applied Chemistry (IUPAC), has never defined the term ‘heavy metal’ it has been widely used in the scientific literature over the past several years. HMs classification in scientific considerations is strongly inconsistent and it takes into account various physicochemical criteria of particular elements, such as density, atomic mass or number, reaction with dithizone, or density for radiation screening [9,12,13]. There is also a tendency to assume that HMs demonstrate ecotoxic or toxic properties. Nevertheless, the definition considering biological harmfulness seems to be misleading and rather imprecise while taking into account the dual role of some metallic elements in plant metabolism. Such conspicuous ambiguity of HMs categorization has led to general confusion regarding the significance of this term that has been recently subjected to a broad discussion. As a result of this consideration, the use of ‘trace element’ rather than ‘heavy metal’ seems to be more justified [14,15,16]. The present review fits well to this novel approach, since it is mostly focused on the profitable impact of the so-called HMs on plant organism. Among them, metallic elements that are specific for serpentine (i.e., Ni, Cr, Co), calamine (i.e., Zn, Pb, Cd), and Cu-containing soils are primarily mentioned, because they may contribute to the formation of highly specialized assemblages of vegetation that tolerate and accumulate potentially toxic amounts of ions. At the same time, these TEs may play an advantageous role in activating the defense pathways in plants that were exposed to various abiotic stress factors. Consequently, in our review, the designation ‘TEs’ instead of ‘HMs’ is applied in order to avoid the instinctive association of these elements with injuriousness, which depends on the applied metal concentration as well as the organism ability to repair nascent damage and acclimatization to emerging adverse conditions.

Many researchers have previously demonstrated the multifaceted importance of metallic TEs in higher plants. Examples include the gene regulation [17], cell signaling [18], secondary metabolites formation [19], CO_2_ assimilation and stomatal conductance [20], and transport across vascular bundles [21]. Therefore, the biological role of each TE is out of discussion in the present review that concerns the recent findings only on elements that are considered as heavy metals or metalloids. The mentioned molecules may contribute to the environmental contamination either by natural or by anthropogenic activities. Among them, both necessary (Co, Cu, Mn, Mo, Ni, Zn) and not required (As, Cd, Cr, Pb, Se) elements for plant metabolism have been discussed. Although a lot of research has been completed in this regard, our study attempts to compile the current data on TEs toxicity for organisms from taxonomically diversified groups, but it highlights the beneficial effects of the metallic ion treatment on plants under stress conditions that provoke the enhanced formation of reactive oxygen species (ROS). Thus, special attention is paid to the involvement of TEs in the oxidative stress mitigation by the activation of antioxidant machinery. What is more, we have emphasized the phenomenon of low-dose stimulation that is caused by non-essential, potentially poisonous elements, called hormesis, which is recently one of the most studied issues. Finally, we have comprehensively reviewed the adaptive traits that are responsible for specialized uptake, accumulation, and detoxification of metallic ions that have resulted in the development of metal-tolerant flora on HM-enriched areas. Taken together, our paper presents the critical approach for metallic element toxicity, which is strongly dependent on dose and genotype.

## 2. Harmful Effects of TEs and Their Consequence on Plant Organisms

Adverse and beneficial responses to TEs are both related to the possibility of metal uptake by organisms. Therefore, the identification of mechanisms that are involved in this process seems to be crucial for understanding the plant reaction to essential and nonessential metals. Since soil is the main source of TEs, their accumulation by plants depends on ions bioavailability for roots [22]. Three categories of TEs can be recognized while taking into consideration that metallic ions exist in the soil as inorganic and organic components, or as precipitates and insoluble compounds: readily bioavailable (Cd, Ni, Zn, Se), moderately bioavailable (Cu, Co, Mn, Mo), and least bioavailable (Pb, Cr, As) [4].

### 2.1. Possible Pathways of TEs Uptake by Plants

Most metallic elements commonly enter plants by roots as simple cations (Zn^2+^, Cd^2+^, Mn^2+^, etc.) from the solution soil phase. Such inorganic forms are taken up much easier and faster than their organic compounds. This uptake of toxic elements involves mechanisms that are analogous to those that are responsible for essential nutrient adsorption [2,23]. Thus, various transporter proteins that are engaged in nutritional ions’ uptake may also help toxic TEs transport across the plasma membrane. There is considerable evidence available that Zn and Cd share transporters of zinc-regulated transporter proteins (ZIP), while As and Se can enter plant roots via the transporters of chemically similar macronutrient phosphate and sulphate, respectively [24,25,26]. Within root tissue, TEs can be translocated towards storage tissue by two parallel pathways—the apoplastic or symplastic [27]. The first way involves passive transport through cell walls and spaces between individual cells, while the second one requires active transport across the membrane. In this case, several other classes of metal transporters that are located in both plasma membrane and tonoplast play a pivotal role in regulating the metal content within the protoplasts. Among them, heavy metal transporting ATPases (HMA), the natural resistance-associated macrophage proteins (Nramp), the cation exchangers (CAXs), the cation diffusion facilitator (CDF), or ZIP family transporters could be mentioned [28,29,30].

As a consequence of non-essential elements similarity to essential ones, the relationship between these two groups may have a negative impact on plant nutrient status under metallic stress conditions. In general, high Zn amount increases the accumulation of other TEs, such as Cu and Mn, in plant organs, but it can reduce phosphorus (P) uptake, manifesting as the purplish red color of leaves [4,21]. A decrease in Zn, Cu, Fe, and Mn content in plant tissues may result from Pb application [12], whereas an excess of Cd ions have shown interference with the accumulation and translocation of P, K (potassium), Mg (magnesium), Zn, and Fe [2,31]. Similarly, a high level of Co and Mn induces Fe deficiency, leading to chlorosis of younger leaves. Further, Co ions modify the transport of P, S (sulfur), Mn, Zn, and Cu from roots to shoots [23]. In turn, high Cu level diminishes the root-to-shoot transport of Fe, Mg, K, P, and Ca, and thus may contribute to changes both in plant morphology and physiological processes, like gas exchanges, photosynthetic rate, and water homeostasis, since these elements are required for the proper functioning of cellular metabolism [32]. On the other hand, such an antagonistic relationship between various elements can be used as a simple stress mitigation strategy that is directly connected with a decline of metal accumulation in plant tissue, but also with indirect beneficial effects of nutrients on activating defense mechanisms against TEs toxicity.

Apart from root uptake, TEs can also be accumulated in above-ground parts by foliar transfer after the deposition of atmospheric particles on the leaf surface. Foliar absorption has been primarily investigated for essential metals, such as Zn, Mn, Cu, Fe, and referred mostly to edible vegetables, like *Solanum lycopersicum* [1], *Spinacia oleracea* [33], and *Brassica oleracea* [34]. Recently, increasing attention is paid to toxic elements, like As, Cd, and Pb, which might be distributed on a large scale with dust particles, and thus pose a health risk for populations that live in industrial and huge urban areas [5,27]. Unlike root metal uptake, which has been largely studied, the relationship between particle accumulation on the leaf surface and their transfer within the tissues still remains poorly understood. What is more, a majority of the research is focused on the concentration and penetration of ions, rather than on pathways that are involved in their distribution inside leaf tissue. The transfer of metal-containing particles depends on various factors. One of the most important is the plant leaf features, such as shape and size, the thickness of epicuticle, as well as the number, size, density, and openness degree of stomata [34]. According to Sgrigna et al. [35], the enhanced air turbulence around the leaves that resulted from a higher leaf area index causes the increase in the dry deposition of contaminated particulate matters. In turn, Xiong et al. [10] showed that the metal-enriched particles firstly adhere to the leaf surface as aggregates, and they are then partly trapped by the epicuticular waxes and partly penetrate in the leaf tissue through stomata. Subsequently, Pb, Cd, and Cu ions from directly exposed leaves are transferred to the newly formed leaves and roots. Similarly to root uptake, foliar absorption of TEs also occurs in a dose-dependent manner. For example, Gajbhiye et al. [36] reported a significant positive relationship between Pb and Cd contents in the leaves of *Cassia siamea* from six heavily polluted areas in Bilaspur, India. What is more, the seasonal variations may control the foliar absorption of contaminants. As stated by Karmakar and Padhy [9], wet foliar surfaces, occurring in the foggy condition and winter season, may capture more dust than the dry leaf areas, however too strong rain can wash the leaves, and therefore the foliar penetration of TEs can decrease.

### 2.2. The confrontation of Root and Foliar Pathways in TEs Interaction with Plants

**Figure 1:**
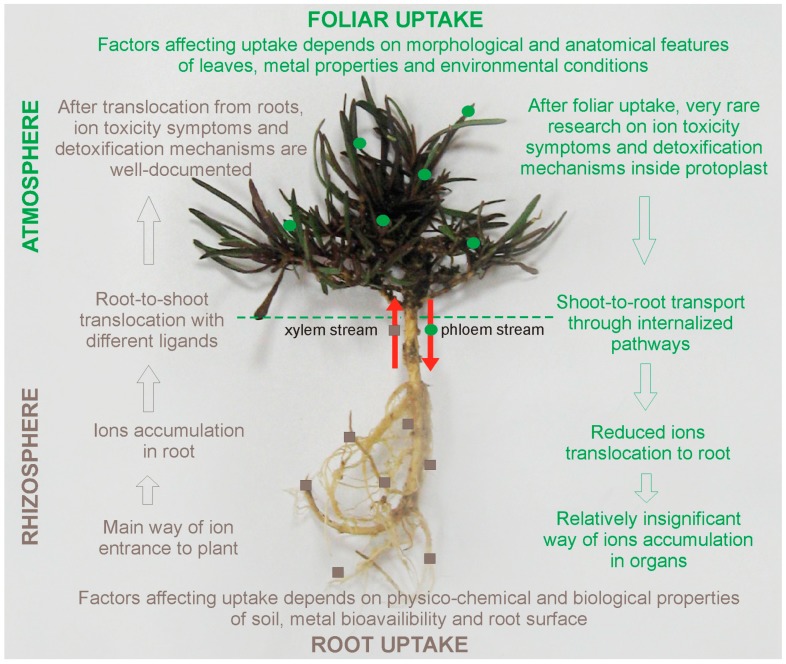
Comparison of pathways that are involved in metallic elements uptake and distribution in plants. Modified from Shahid et al. [37]. Photo of *Gypsophila fastigiata* calamine ecotype provided by E. Muszyńska.

Although TEs can be accumulated in plants through both root and/or foliar uptake, mechanisms that are involved in their absorption, translocation, and compartmentation differ, depending on the pathways of metal application (Figure 1).

Roots are the major ways by which ions enter the plants. Therefore, the physico-chemical properties of soils, such as pH, organic matter content, cation-exchange capacity, and microbial activity significantly modify TEs bioavailability to plant roots [11,38,39]. In turn, the type and chemistry of TEs, as well as the characteristics of the plant leaf surface and plant maturity, may affect foliar uptake [5,34]. What is more, approximately 95% of the toxic elements (mostly Pb ions) absorbed by roots is stored within their cells, unless the plant is able to hyperaccumulate TEs or it is treated with chelators improving ions accumulation in shoots [39]. The reduced root-to-shoot transport results from: (1) the presence of Casparian strips in the endodermis, which play a role of physical barrier regulating water and ions movement to the stele, (2) precipitation in the intercellular space as insoluble metal-salts, or sequestration in the vacuoles of cortex cells [40,41,42]. Similarly, after foliar deposition, a majority of the TEs remain in the organ by which they are absorbed and only less than 1% of ions is transported to root tissues and other organs [10,43]. However, the mechanisms preventing the translocation of leaf-absorbed TEs within the organism still remain unknown. In the case of TEs distribution inside the plants, the movement of root-absorbed ions to aerial parts takes place via the xylem stream and it is mostly governed by the transpiration rate [44]. Moreover, a number of important membrane transporter gene families, that participate in ion translocation to the above-ground parts of plants, have been recently recognized [28,45,46]. In contrast, the transport of elements taken by leaves runs through phloem stream towards other plant parts and there is no clear evidence on the potential role of various chelators or genes that are involved in this movement (Figure 1). The complication of research on metal distributions after foliar uptake may be associated with difficulties in distinguishing of TEs translocation pathways, since ions presence within aerial tissues may result from either its root-to-shoot transport or from direct leaf exposition. Thus, future research is needed on diversified plant species and applied ions, as well as their transfer at tissue and cellular level. Undoubtedly, the better understanding of particle-phyllosphere interaction may contribute to an improvement of life quality on heavily polluted and urban areas.

### 2.3. Structural and Metabolic Modifications under Metallic Elements Exposure

Toxic ions and/or their toxic concentrations may contribute to the multiple disorders in plants resulted not only from the direct impact of these elements on numerous processes, but also from additional secondary consequences of oxidative stress, regardless of exposure type (root or shoot). Studies on ions toxicity after foliar uptake and defense mechanisms against stress are less elucidated than after root uptake despite clear evidence about processes modifications after TEs treatment, and contrasting reports about the positive, negative, and neutral impact on plants suggest the complexity of this phenomenon [47,48].

Abundant studies have shown that metallic ions may induce the disintegration of cell organelles. A reduction of chloroplasts number and size, accompanied by a looser arrangement of thylakoids and grana, were noticed in many aquatic and terrestrial plants under Cd, Cu, Cr, Ni, Pb, and Zn treatment [49,50,51]. These disturbances in chloroplast ultrastructure negatively affect the photosynthetic pathways, while the decomposition of mitochondria may greatly hinder plant intracellular respiration [52,53]. The elevated concentrations of TEs adversely change the nuclei ultrastructure, which causes the nuclear envelope destruction or nucleus volume reduction, chromatin condensation, chromosomal aberrations, and nucleic acids damage, resulting in decreased cell mitotic and transcriptional activity [34,54,55]. In turn, the disruption of plasma membrane integrity was ascertained, among others, in *Erythrina fusca* [32] or *Lemna gibba* [56] under Cu, Cr, and Cd stress, whereas malformations of the cell walls were found in *Brassica napus* cultivars that were treated with Cr [52] or Co ions [57].

Apart from cellular damage, the negative impact of TEs on physiological status has been also well documented. It is not surprising that the plant responses to TEs actions vary between particular taxonomical groups. In Table 1 the arbitrary selected findings on biological alterations that may occur in differentiated taxa from Cyanobacteria to vascular plants under TEs stress conditions are briefly summarized. In general, the deleterious direct effects of TEs include the disturbances in gaseous exchange, respiration, and CO_2_ fixation [3,20]. It was found that an excess amount of TEs on the photosynthetic apparatus is manifested in the degradation of enzymes involved in chlorophyll biosynthesis, decreased chlorophyll and carotenoid contents, changes within reaction center, photosystem II and photosynthetic rate, as well as disturbances in the transport of electrons between the antenna system and photosystems [50,53,58,59,60,61,62,63,64]. Furthermore, the elevated concentration of TEs may lead to the inactivation of key enzymes of various metabolic pathways, as well as blocking the functional groups of metabolically important molecules. As an example, the significant decrease in activities of nitrite reductase, nitrate reductase (EC 1.7.1.2), nitrogenase (EC 1.18.6.1), glutamate dehydrogenase (EC 1.4.1.2), and glutamine synthetase (EC 6.3.1.2) [65], as well as many antioxidant enzymes have been reported [66,67,68,69,70]. As a consequence of being closely related to other metabolic and ultrastructural changes, plants under metallic elements stress demonstrate visible symptoms of toxicity. They may appear at various stages of plant life cycle, and they include the retardation of seed germination and early seedling development, shoot and root growth inhibition, leaf chlorosis and necrosis, or even organism death [7,55,71].

The common consequence of toxic metal treatment is the enhanced and uncontrolled release of ROS that disturbs the normal balance of O_2_^•−^, ^•^OH, and H_2_O_2_ in the intracellular spaces [82]. The predominant ROS sources include disturbances of electron transfer in chloroplasts and mitochondria, as well as oxidative metabolism in the peroxisomes [83,84]. Additionally, plasma-membrane bound NADPH oxidase may contribute to the formation of O_2_^•−^ molecules [85].

ROS production might be stimulated by either TEs redox activity or by direct effects of toxic ions on metabolism in a subcellular site-specific manner [86]. Thus, the over-generation of ROS in the presence of TEs can occur as a result of:

(1) single-electron reactions of redox-active metals, such as Fe, Cu, Co, Ni, and Cr, which are involved in the formation of ^•^OH from H_2_O_2_ and O_2_^•−^ via the Haber–Weiss and Fenton reactions (for example Fe^2+^ ↔ Fe^3+^ + e^−^ and Cu^+^ ↔ Cu^2+^ + e^−^);

(2) disturbances of metabolic pathways that increase the rate of ROS formation; and,

(3) diminutions the efficiency of antioxidant machinery by the depletion of low molecular weight antioxidants, as well as the inactivation and/or down regulation of antioxidant enzymes activity caused by both redox-active and inactive metals (Pb, Cd, Hg) [87].

ROS are described as ‘double-edged sword’ in plant biochemistry and physiology [88]. On the one hand, oxidative damage of macromolecules appears if the metal-induced accumulation of ROS is not adequately counterbalanced by cellular enzymatic and non-enzymatic antioxidants. The overproduction of these highly active molecules leads to lipid peroxidation, proteins oxidation, nucleic acids damage, membrane dismantling, ion leakage, and programmed cell death [89,90]. On the other hand, ROS are important secondary messengers in plants under metallic stress conditions (for review see [82,91]).

## 3. Second Face of TEs—Beneficial Significance in Abiotic Stress Mitigation by ROS Scavenger Activation

Despite the clear evidence about the harmfulness of TEs, they may also perform several specific and crucial roles in plant protection against various abiotic stresses, such as salinity, toxic concentration of metallic elements, chilling, high temperature, drought or excess water, and light. A common plant reaction to unfavorable conditions is the enhanced ROS production, regardless of stress factor. Scientific research from the latest decades clearly demonstrates that mitigating oxidative stress by TEs is the most studied issue. Results that are presented by many authors suggest that the disturbance of redox homeostasis switches on signalling pathways in cells, resulting in the acclimatization to the conditions that are triggered by abiotic stresses. In consequence, the oxidation of cell molecules is alleviated or even completely inhibited. Therefore, TEs can activate the defense mechanisms that may simplify plants that exist under stress conditions. As an example, Rahman et al. [92] provided results regarding the response of rice plants to salt stress and the possibility of mitigating its effects through the application of Mn ions to the hydroponic growth medium. It was observed that the solution containing 0.5 mM MnSO_4_ stimulated ROS scavenging systems in plants that wre treated with 150 mM NaCl, as was reflected in increasing the content of non-enzymatic antioxidants (flavonoid and phenolic molecules and reduced ascorbate), enhancing the activity of enzymes, such as superoxide dismutase (EC 1.15.1.1, SOD), catalase (EC 1.11.1.6, CAT), dehydroascorbate reductase (EC 1.8.5.1, DHAR), and monodehydroascorbate reductase (EC 1.6.5.4, MDHAR). Methylglyoxal neutralisation mechanisms comprising induced activities of glyoxalases was also observed. Consequently, *Oryza sativa* plants could return to proper growth and Mn treatment contributed to partial recovery from the inhibited growth and physiological disturbances (e.g., chlorosis) by ameliorating ion and osmotic equilibrium through diminishing Na^+^ influx and enhancing water balance, respectively. Therefore, the beneficial effect of Mn was expressed in better salt stress overcoming via the modulation of ion homeostasis and oxidant and methylglyoxal detoxification mechanisms.

For ROS scavenging, plants use two antioxidant mechanisms—non-enzymatic and enzymatic. The latter one is based on the activity of several antioxidant enzymes, among which SODs seem to play a particularly important role. SODs are oxidoreductases that perform a fundamental function in ROS detoxification responses. In the effect of SOD activity, the amount of superoxide anion and H_2_O_2_ molecules is controlled and the cells are protected from the effects of oxidative stress. The study of Muszyńska et al. [93] on three different ecotypes of *Silene vulgaris* originated from the non-contaminated area (non-metallicolous ecotype) and from calamine or serpentine waste heaps (two metallicolous ecotypes) revealed enhanced SOD activity in both metallicolous ecotypes. While taking into account the elevated level of total Zn (about 10,690 mg kg^−1^) in the substratum on which plants of calamine ecotype have grown, and toxic amount of Ni ions (1300 mg kg^−1^) ascertained in serpentine substratum, the correlation between this enzyme activity and the type of TEs in the soil and/or the plant genotype suggests the potential role of Zn and Ni in the antioxidant machinery of metal-tolerant specimens. Schickler and Caspi observed the analogous relationship [94] among *Alyssum* genus in the hyperaccumulating species—*A. argenteum* and *A. maritimum* that were exposed to Ni or Cd ions. It is well known that some TEs are key molecular components necessary for the proper functioning of enzymes; therefore their amounts in plant organisms are tightly controlled and regulated. The SOD occurs in three isoforms in plants, so the activity of MnSOD (Mn cofactor), FeSOD (Fe cofactor), and Cu/ZnSOD (Cu and Zn cofactors) is presented. SODs are regulated on the transcriptional and translational level in multiple mechanisms. Herald et al. [95] proved that *Arabidopsis thaliana* plants that were treated with Cu, Fe, or Zn showed an increased level of Cu/ZnSOD transcripts. Correspondingly, the high level of Ni and Co in serpentine soils and Zn in calamine ones contributed to a higher concentration of these TEs in *Silene vulgaris* organs, which may lead to the stimulation of TEs-dependent activity of enzymes [93]. These findings are in accordance with López-Millán et al. [96], who demonstrated a linear increase in Cu/ZnSOD activity with an increased level of Zn in *Medicago truncatula* specimens growing hydroponically under different Zn supply in the nutrient solution. Although the above-mentioned results suggest that the activity of different SOD isoforms may be stimulated by different elements, their common role is based on better protection against oxidative stress.

Glyoxalases (GLYs), including glyoxalase I (EC 4.4.1.5, GLYI, S-D-lactoylglutathione lyase) and glyoxalase II (EC 3.1.2.6, GLYII, S-2-hydroxyacylglutathione hydrolase) [97], are not as widely known enzymes as SODs, which participate in plant response to stress conditions. Glyoxalase I uses Ni as a cofactor and it metabolizes highly cytotoxic metabolite methylglyoxal to S-lactoylglutathione with the participation of reduced glutathione molecules. Furthermore, glyoxalases catabolize various carbonyl compounds, such as α-oxoaldehydes (glyoxal and hydroxy-pyruvaldehyde), which can be accumulated in plants under stress conditions [98,99]. The analysis of glyoxalase isoforms in rice that was conducted by Mustafiz et al. [99] demonstrated that one of them, called OsGLY11.2, is activated by Ni^2+^ and *OsGLYI-11.2* transgenic tobacco plants exhibited tolerance against osmotic, methylglyoxal, oxidative, and salinity stresses. These findings clearly show the beneficial role of Ni in plants growing under abiotic stress conditions.

Wu et al. [100] provided interesting results that shed new light on the understanding of the TEs beneficial effect on plant growth during stress. These authors verified the hypothesis that Mo participates in nitric oxide (NO)-induced antioxidant enzyme responses to drought stress in winter *Triticum aestivum* through Mo-dependent enzymes, especially by nitrate reductase, which uses Mo as the cofactor. It was found that Mo supplementation in Hoagland solution enhanced mRNA contents and antioxidant enzyme activities in the leaves of wheat during drought stress. Simultaneously, the amount of hydrogen peroxide and lipid peroxidation markers (thiobarbituric acid reactive substances, TBARs) were lower than in the specimens that were untreated with Mo. Together with the stimulation of antioxidant mechanisms, elevated NO production was noted, which suggested that the Mo-stimulated antioxidant response may be related to NO activity. Furthermore, NO scavengers and Mo-enzyme inhibitors caused a reduction of Mo-induced activity and mRNA levels of antioxidant enzymes and, on the other hand, the NO donors alleviated these inhibiting effects. Additionally, it was also proved that Mo treatment leads to increased activity of nitrate reductase in leaves of *T. aestivum* during drought stress, which implies that this enzyme may participate in the production of NO induced by Mo treatment. Therefore, it can be supposed that NO regulates the induction of antioxidant mechanisms during abiotic stresses and that nitrate reductase is involved in these processes. Wu et al. [100] showed that Mo can alleviate the negative effects that are caused by drought stress, whereas Ma et al. [101] noticed the similar influence of Zn application on wheat plants exposed to drought. Zn treatment under drought stress enhanced the accumulation of non-enzymatic antioxidants, such as reduced glutathione and reduced ascorbate, phenolic, and flavonoid molecules in wheat flag leaves. Apart from the enhanced accumulation of antioxidants, the levels of antioxidant enzyme transcripts increased in relation to SOD, CAT, glutathione reductase, monodehydroascorbate reductase, dehydroascorbate reductase, and ascorbate peroxidase (EC 1.11.1.11, APX), as well as flavonoid synthesis—chalcone synthase (EC 2.3.1.74, CHS) and phenylalanine ammonia-lyase (EC 4.3.1.24, PAL). What is important, the H_2_O_2_ content and the intensity of lipid oxidation processes were reduced at the same time, while the parameters of chlorophyll *a* fluorescence and chlorophyll content significantly increased. Thus, it can be concluded that Zn is one of the pivotal micronutrient players during plant abiotic stress alleviation that significantly alters processes that are related to the keeping of plant redox homeostasis. This claim is also confirmed by the research of Ammar et al. [31]. The tomato plants simultaneously treated with Cd and Zn ions showed a significant decrease in the lipid peroxidation level and lipoxygenase (EC 1.13.11, LOX) activity, whereas these stress parameters were induced by the separate application of Cd [31]. Curiously, Zn has been also able to repair lipid content and composition, which was expressed in the enhancement accumulation of total lipids, phospholipids, and galactolipids, as well as neutral lipids.

Osmotic stress is another abiotic stress on which plants can suffer. The preventive activity of Co on potato plants under osmotic stress that is triggered by polyethylene glycol 4000 was noted by Li et al. [102]. It has been noted that the cell membrane damage, lipid peroxidation, and the depletion in chlorophyll content that was induced by osmotic stress in leaves were abolished due to the presence of Co ions in testing solution. Additionally, Co treatment alleviated the decrease in putrescine, spermidine, and spermine amounts and the activities of SOD, CAT, APX, and guaiacol peroxidase (EC 1.11.1.7, POD), hence contributing to the prevention of accumulation of H_2_O_2_ and superoxide molecules.

Conducting research that aimed at understanding the plant molecular mechanisms responsible for stress tolerance is important not only for better acquainted with the biology of the analysed species, but it also may be applied in horticultural and agricultural production in order to increase the field yields of good quality. As an example, the experiments of Gómez-Muñoz et al. [103] suggest that the the Mn and Zn application can alleviate the effects of low-temperature stress in maize that was planted in fertile soil and lead to obtain an increased biomass yield of leaves at 51 days after sowing in comparison with the untreated control plants. Taken together, Co, Cu, Fe, Mn, Mo, Ni, and Zn play essential roles in normal plant physiological processes, but adverse environmental conditions can disrupt the functioning of plants. The mentioned TEs succour plants under stress by several molecular and physiological mechanisms, including (1) the stimulation of non-enzymatic and enzymatic antioxidant machinery, (2) activation of methylglyoxal detoxification, (3) keeping the redox, ionic, lipid, and polyamine balances, as well as (4) maintenance of the photosynthetic efficiency and stable amounts and composition of photosynthetic pigments. Thus, the optimum supplementation with beneficial TEs can facilitate and promote plant growth and development under both normal and stress conditions.

## 4. Hormesis Effect

The involvement of some essential TEs in the mitigation of various stress effects fits well to the hormesis concept, which refers mostly to ions with unknown physiological function in scientific literature. Hormesis emphasizes that the influence of metallic elements on plant organisms depends on metal concentration. Some of them may lead to strong growth inhibition or even organism death when they are applied in high doses, while the low concentrations of the same substances may have beneficial effects. This biphasic dose–response phenomenon in plant research has been firstly observed by Shultz at the beginning of the 19th century [104]. Initially, the hormesis concept was marginalized until the last several decades, when it has attracted considerable attention. The research of Agathokleous [105] revealed that more than 2400 scientific articles from the years 1900–2016 included one or more of keywords that were connected with hormesis, and unsurprisingly 91% of them have been published after the year of 2000. Most of the experiments are being conducted toward assessing the dose–response issues on pesticides, phytotoxins, UV, or ozone treatments, and their underlying mechanisms [106,107,108]. Nevertheless, the hormetic response has been also observed in plants that were exposed to low concentrations of non-essential metal ions (Cd, Pb, Cr), non-metallic trace elements (As, Se), representatives of the platinum group (palladium Pd, platinum Pt), or even rare earth elements (lanthanum La). The stimulatory impact of potentially toxic elements that were applied at low-concentration referred to the improvement of many morphological and physiological traits [12,67,109,110]. For example, the hormesis effect of Cd on the growth and yield was observed in *Solanum melongena* [111] and *Brassica napus* [112], whereas the increase in shoot and root growth, biomass accession accompanied by the enhanced photosynthetic pigments accumulation, and photosystem II functioning were noticed for *Dianthus carthusianorum* [12] and *Lonicera japonica* [67,68]. Similarly, *Weigela florida* ‘Red Purple’ did not show visible symptoms of Cd toxicity at its low dose, and even the stimulation of growth and antioxidant enzymes activity (SOD and POD) were ascertained, followed by their gradual decline at higher concentrations. In turn, *Miscanthus sacchariflorus* showed hormetic response under simultaneous treatment with Cd and As with respect to chlorophyll concentration and the net photosynthetic rate [80]. The same elements, but applied separately, caused a significant rise in photosynthetic pigments’, total protein, and non-enzymatic antioxidant content in *Spirodela polyrrhiza* [81]. A hormesis was also recognized for Pb ions in the case of *Anthyllis vulneraria* [53] or *Pisum sativum* [110]. Besides, the adventageous effect of Cr application on biomass and/or photosynthesis efficiency was shown for taxonomically diversified species, such as *Allium cepa* [113], *Jatropha curcas* [114], *Theobroma cacao* [115], and *Lemna minuta* [116]. Further, *Astragalus cicer* and *A. bisulcatus* responded positively on Se dosage manifesting in the aboveground mass increase [117]. A similar increase in biomass production and in the root-shoot ratio was reported in *Symplocos paniculata* [118], whereas growth improvement occurred concomitantly in *Camelia sinensis* with an enhanced rate of net CO_2_-assimilation, chlorophyll *a*, and carotenoids content, as well as the concentration of carbohydrate and free amino acid compounds in young leaves [119]. Progressively, hormetic response also has been recognized for unusual elements, such as Pt, which positively influenced photosynthesis efficiency and non-enzymatic antioxidant accumulation in *A. thaliana* plants [120]. As reviewed by Agathokleous et al. [105], low doses of La may also induce beneficial reaction in many species—*Vicia faba*, *Glycine max*, *Oryza sativa*, and so on.

**Figure 2:**
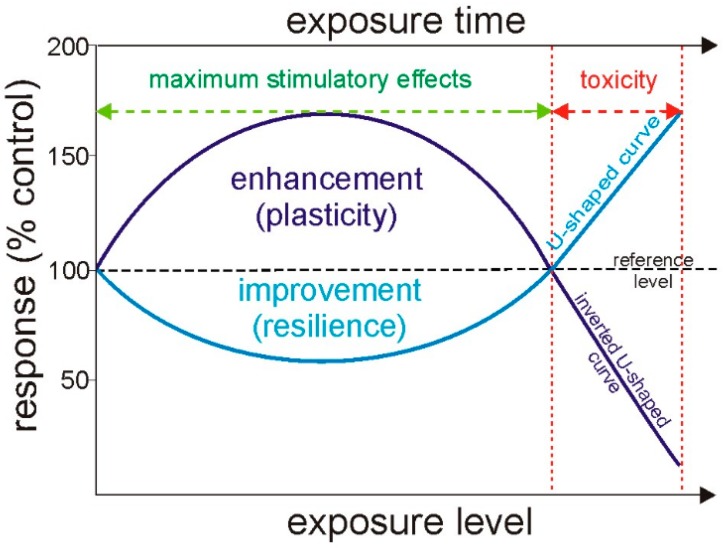
Graphical presentation of the hormesis concept. Dose-response curves showing the changes in biological responses to treatment (exposure level) with time (exposure time) in relation to a reference group (% control). Response that may refer to both beneficial and deleterious effects is calculated according to the formula: response = *p*_c_/*p_t_* × 100, where *p_c_* is the mean value of the tested parameter (*p*) in the control group and *p_t_* is the mean value of *p* in the treated group expressed in percentage. The regions of enhancement/improvement as well as adverse or toxic effects are also presented. Enhancement (plasticity) is the ability of organism to survive by the acclimatization to nascent conditions, while improvement (resilience) refers to organism recovering by quick repair of appearing damage. Modified from Agathokleous [105].

The hormesis might be graphically illustrated by dose-response curves, as shown in Figure 2. The most common form is the so-called β- or inverted U-shaped curve, which depicts low-dose enhancement and high-dose inhibitory reaction (Figure 2). On the contrary, J-shaped or U-shaped curve describes the low-dose reduction and high-dose stimulation [121]. Regardless of curve type, its amplitude is limited, and a maximum stimulatory response below two-fold of controls and a width lower than 10-fold in a dose range can appear [122].

According to Calabrese and Baldwin [123], the growth stimulation is due to ‘an adaptive compensatory process following an initial disruption in homeostasis’. This hypothesis seems to be highly attractive in view of the current knowledge regarding stress signaling cross talk. Recent experiments of Moustakas et al. [109] on seagrass *Cymodocea nodosa* suggested the influence of Cu nanoparticles on the induction of stress defense mechanism through H_2_O_2_ production. These findings are in accordance with the study on *Silene vulgaris* ecotypes that were treated with Pb or Ni ions, which revealed H_2_O_2_ accumulation in leaves, even before toxic elements got into the aerial part of plants [51]. Thus, metal ions can act as elicitors of defense mechanism that, in turn, can facilitate the plant growth and development, particularly under stress conditions [124]. It could be confirmed by extensive research on preconditioning protection by biotic and abiotic factors against the subsequent higher exposure [106,125,126]. Therefore, the hormetic response seems to be a general strategy, by which cells, organs, and organisms acclimate to more severe challenges, leading to the speciation in the evolutionary process [127].

## 5. Metallophytes as Unique Communities from Metalliferous Sites

The presence of elevated concentration of metallic TEs in the soil contributes to (micro)evolutionary changes that have resulted in the development of adaptive traits in plants, enabling them to survive and reproduce in harsh edaphic environments [7,128,129]. Upon time, anthropogenically created metalliferous areas are spontaneously colonized by plant species or specialized ecotypes that occur on both polluted and unpolluted terrains. Such so-called pseudometallophytes (or facultative metallophytes) have developed metal tolerance in a relatively short time of 40–150 years or even less than a decade [130]. In turn, the obligate metallophytes live only on metal-enriched soils by mineral deposits and they exhibit more specialized mechanisms of tolerance that have been built for centuries [129]. The evolution of metal tolerance is accompanied by an increased requirement of tolerant specimens for higher amounts of metal(s) to which they have been adapted, according to Antonovics et al. [131]. It could be reflected in better growth and the development of specimens that are adapted to metalliferous habitats than specimens of the same species, but originating from unpolluted sites during exposition to the same metal level, as observed in *Biscutella laevigata* [128], *Dianthus carthusianorum* [132,133], or *Armeria maritima* [134]. It is also probable that mechanisms of ion detoxification in metal-tolerant plants are so efficient that the threshold of toxicity in these specimens is much higher than in other ones, thus they show growth disturbances later or not at all [135]. Both of these explanations fit well hormesis concept and certainly indicate the important role of TEs in metallophytes metabolism.

**Figure 3:**
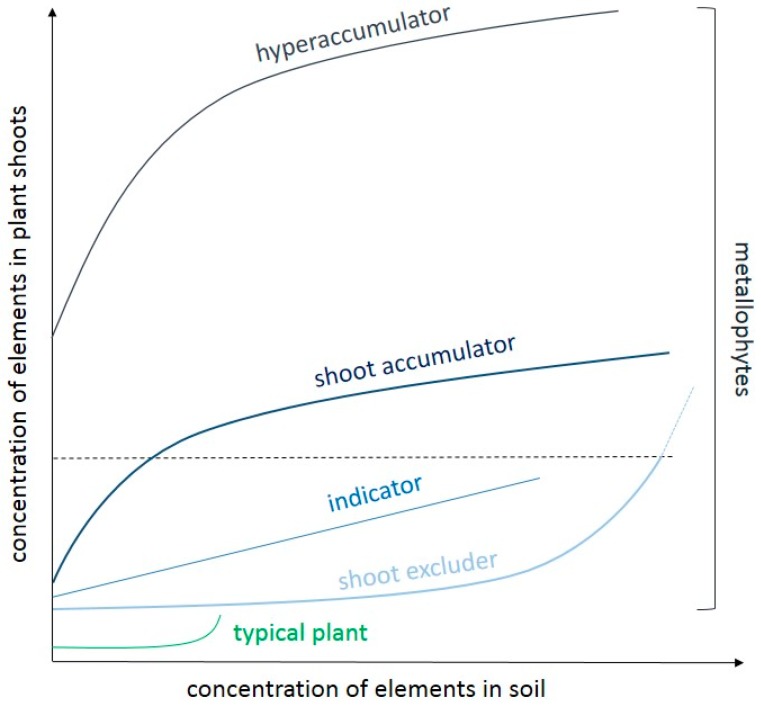
Conceptual response diagram demonstrating the potential relationship between the concentration of metals/metalloids in aerial parts of the plant and available content of metals/metalloids in the soil. Modified from van der Ent et al. [14]. The dotted line showes the hyperaccumulator threshold for the different metallic.

Particular metallophytes considerably differ in their ability to metal uptake, and thus two types of relationship between elements content in soil and plant tissue can be recognized: exclusion and accumulation (Figure 3).

The simplest strategy is to avoid the metal uptake from the soil or to minimalize ions movement inside the organism (Figure 3). Therefore, the majority of metallophytes can be classified as ‘excluders’ that reduce either ions uptake by roots or their translocation from underground tissues to the aerial ones. The modification of rhizosphere that contributes to a decrease in metals bioavailability plays a significant role in avoiding metallic ion penetration. Excluders are able to secrete into the soil organic acids (like citrate, malate, or oxylate) as well as amino acids (like histidine) that chelate metallic ions and transform them into insoluble forms. The restricted metals uptake by root exudates was observed in different species, among others *Thlaspi arvense* [136], *Ricinus communis* [137], or *Miscanthus sacchariflorus* [138] that were treated with Ni, Cu, and Cd ions, respectively. An analogous role is performed by phytosiderophores that are produced by roots of monocot plants to limit the acquisition of Fe ions under their excess amount, as observed e.g., in *Triticum aestivum* [38]. Other mechanisms involve the myccorhizal fungi that may reduce metal uptake by altering the soil metal bioavailability or sequestering metals in their own tissues [139,140]. Besides the changes in the environment around the roots, their surface that is covered by mucilage enriched with uronic acid constitutes an effective barrier against the penetration of TEs [141]. Furthermore, root cell walls that were composed of cellulose, hemicellulose, pectin, and proteins provide many hydroxyl or carboxyl groups that bind ions and thus decrease their migration into the protoplast [3,40]. Cell walls can immobilize about 80–90% of Pb, Cd, Cu, or Zn, as reported for many plant species. For instance, Pb- or Cd-complexes with root cell wall components were observed in *Arabidopsis thaliana* [41], *Dittrichia viscosa* [142], or *Dianthus carthusianorum* [40], while Zn deposits were abundant in the root cortex cells of *Solanum nigrum* [143]. It is also noteworthy that the structure of cell walls may be actively remodeled under the exposure to elevated TEs level by thickening and increasing the content of the low-methyl esterified fraction of pectins, as ascertained in the terrestrial herb *Arabidopsis thaliana*, tree *Populus tremula x tremuloides*, and aquatic plant *Lemna trisulca* treated with Pb ions [144] or *Solanum tuberosum* growing in the presence of Cd and Zn [141].

**Figure 4:**
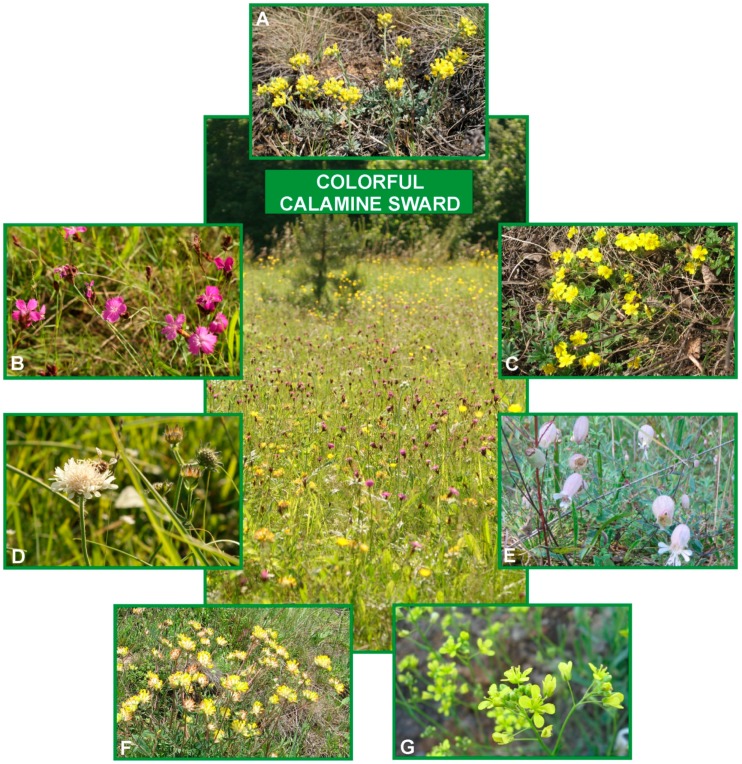
Vegetation cover on more than one hundred years old calamine waste heap in Olkusz Ore-Bearing Region (southern Poland). Examples of metallophytes with excluder behaviour: *Alyssum montanum* (**A**), *Dianthus carthusianorum* (**B**), *Potentilla arenaria* (**C**), *Scabiosa ochroleuca* (**D**) or *Silene vulgaris* (**E**) and commonly known hyperaccumulators: *Anthyllis vulneraria* (**F**), and *Biscutella laevigata* (**G**). Photos by E. Muszyńska.

Another exclusion mechanism is based on the maintenance of low, constant concentrations of TEs in the shoots. At the cellular level, ions that have penetrated inside the root protoplasts are detoxified by chelation in the cytoplasm or storing into vacuoles [145]. In turn, the endodermis layer protects from ions entrance to vascular bundles and then translocation to the shoots at the tissue and organ level [40]. However, it should not be forgotten that, despite many processes that regulate ions accumulation and transport in plants, when metal concentrations in the environment reach the critical level, the control mechanisms that are responsible for ion homeostasis in the protoplast may break down and the unrestricted influx of cations occurs. Among the plants colonizing highly polluted with heavy metals post-industrial waste heaps, excluder behaviour has been observed in specimens from metalliferous populations of *Alyssum montanum* [90] (Figure 4A), *Dianthus carthusianorum* [146] (Figure 4B), *Potentilla arenaria* [147] (Figure 4C), *Scabiosa ochroleuca* [147,148] (Figure 4D), or *Silene vulgaris* [16] (Figure 4E).

The second strategy relies on the active TEs uptake from the soil and their subsequent translocation and accumulation in the shoots (Figure 3). Unlike the excluders, which retain and detoxify most of the taken ions in the root cells, accumulators efficiently translocate these elements to the shoot via the xylem [149]. Several types of transporters have been recognized to be involved in root-to-shoot translocation, among which the P_1B_-type ATPases and the yellow strip like (YSL) family members are of particular importance [150]. Metallic elements are loaded into the xylem and are transported for a long distances with different ligands, primarily with free amino acids, such as histidine and nicotinamine, which form stable complexes with bivalent cations [151]. It has been also shown that organic acids may be responsible for ions loading and unloading into and out of xylem and phloem, as observed in *Sedum alfredii*, in which the citrate concentration in xylem sap increased with increasing Zn content in the growing medium [149]. Additionally, a higher level of citrate and malate accumulation was noticed, for example, in leaves of Ni-hyperaccumulators *Alyssum murale* than in related non-hyperaccumulator *A. montanum* [152] or in calamine ecotype of *Armeria maritima* [134], which indicates the role of these acids in metallic ion chelation. However, the importance of the above-mentioned substances in metal detoxification is questionable and it varies between species, since no significant differences in malate and citrate content were found in the contrasting ecotypes of *Dianthus carthusianorum* [133] or *Echium vulgare* [64] that were exposed to Cd ions. Despite it, in accumulators, the efficient ions translocation from root-to-shoot is associated with the maintenance of the metallic element content in metabolically active cytoplasm of shoot cells at a low level. This is possible due to the sequestration of metals into the vacuoles and cell walls, and their further removal from the organism by older leaves, as in *Biscutella laevigata* or *Anthyllis vulneraria*, and from leaf cells by salt glands, as in *Armeria maritima* ssp. *halleri* [134], or by trichomes as in *Alyssum* sp. [90,153] and even cuticle as in *Arabidopsis halleri* [154].

Metal ‘indicators’ and ‘shoot accumulators’ can be distinguished among the plants that exhibit the ability to accumulate TEs in their above-ground tissue. In ‘indicators’, the concentrations of metallic elements in shoots reflect their external contents due to passive ions adsorption. Such plant species have been proposed for the monitoring of environmental pollution [8]. However, to date, no species have been found to demonstrate a strong positive correlation between soil and shoot metal concentration to polymetallic contamination that is characterized to the most metalliferous sites [129]. Therefore, among the accumulators, the most interesting are ‘hyperaccumulators’ that can accumulate metals and metalloids in their aerial parts at concentrations from 100 to 10,000 mg/kg dry weight, i.e., from 50 to 100-times higher than in non-hyperaccumulators without showing any toxicity symptoms [155].

The criteria that allow for designating a plant as hyperaccumulator are not uniform and have changed since the early reports defining the levels of TEs concentrations in dried leaves. Baker and Brooks firstly proposed the threshold values for particular metals and metalloids [156], however, van der Ent et al. [14] have suggested the new currently applicable levels that are presented in Table 2. Furthermore, the determination of hyperaccumulator status should base only on elements that are accumulated in leaves via active ions translocation inside plant organism; thus passive accumulation via foliar deposition is not regarded as hyperaccumulation. Plants that were obtained from seeds collected in natural conditions could be cultivated on soils from natural habitats in a greenhouse or climate room instead of in hydroponic to avoid airborne contamination and other uncontrolled misstatements [14].

In the Global Hyperaccumulator Database (http://hyperaccumulators.smi.uq.edu.au/collection), approximately 720 species that represent 52 families and 130 genera have been registered so far as hyperaccumulators of metallic elements. It is supposed that this phenomenon has evolved independently many times, while taking into account the wide distribution of hyperaccumulation in angiosperm phylogeny [155]. Most of the plants hyperaccumulate Ni(523), Cu (53), Co (42), Mn (42), Se (41), and Zn (20), but there are also some species showing hyperaccumulation of more than one element [15] (Table 2). Probably the best-known hyperaccumulator is *Noccaea (Thlaspi) caerulescens* that together with *Arabidopsis halleri* are widely used as model plants in research on metal homeostasis and detoxification [145,158,159,160,161]. The extraordinary amounts of TEs have been also reported, among others, in Zn-hyperaccumulator *Anthyllis vulneraria* [160,162] (Figure 4F) and in *Biscutella laevigata*—an unusual hyperaccumulator of Tl (thallium) [163,164] (Figure 4G).

Significant progress in understanding the mechanisms that overlie metal hyperaccumulation has been made through the comparative research of physiological, genomic, and proteomic traits between hyperaccumulators and related non-hyperaccumulating plants [153,155,158,165]. As a result of these considerations, hyperaccumulation phenomenon can be characterized by three general properties: enhanced ability to take up metallic elements by roots, effective translocation of ions from underground parts to aerial ones, as well as the activation of efficient defense mechanisms enabling detoxification and sequestration of huge amounts of metallic ions in the leaves. More detailed biochemical and molecular aspects of this phenomenon and metal tolerance, in general, have been broadly described over the last few decades in many reviews [15,129,157,166,167] and research articles [42,51,93,133,146,168]. Interestingly, the mentioned steps in hyperaccumulation do not depend on novel genes, but they rely on genes that are common to both hyperaccumulators and non-hyperaccumulating relatives; however, they are differently regulated and expressed in these two groups of plants [157]. Despite it, the question regarding the existence of one ‘master gene’ or some transcription factor that coordinate molecular processes that are involved in (hyper)accumulation still remains open.

## 6. Conclusions

There is clear evidence that TEs play a dual role in plant growth and development. Although some of them are necessary for proper metabolism, both essential and non-essential elements may contribute to disturbances of biological functions in elevated concentrations, leading even to organism death. On the other hand, metallic elements may help plants to cope with adverse conditions, and therefore their application can be used as a simple stress mitigation strategy. Synergistic and antagonistic relationships between particular TEs can regulate ion uptake and accumulation in the tissue, as well as encourage the antioxidant defense system. However, the pathways of metal absorption differ for different environmental conditions and for different species, and thus the plant response to TEs exposure can also be diversified. The elements that are toxic for some organisms can be simultaneously beneficial for another one. The occurrence of special metallicolous vegetation on metal-enriched areas could confirm it. Through microevolutionary changes, these unique plant species or specialized ecotypes have adapted to an extreme level of metallic elements in the soils and they exhibit various mechanisms to accumulate and detoxify enormous amounts of ions inside their tissues without suffering from toxicity. In this context, the present review provides a comprehensive approach to TEs relationship with living organisms and demonstrates the novel insights into the metallic stress concept.

## Figures and Tables

**Table 1:** The examples of toxic trace elements (TEs) effects on metabolic and growth processes in chosen representatives of diversified taxonomic groups in alphabetical order within the taxon.

Taxonomic Group	Taxa Examples	Ion(s)	Response		Ref.
			Decrease	Increase	
Cyanobacteria	*Synechocystis* sp. PCC6803	Ni, Cd	phycocyanin α-subunit	-	[72]
		Ni, Co	glucose-1-phosphate adenylyltransferase	-	
		Ni, Co, Cd	ribulose1,5-bisphosphate carboxylase, periplasmic iron-binding protein	-	
		Ni	-	aspartyl/glutamyl-tRNA (Asn/Gln) amidotransferase subunit B, ferredoxin-NADP oxidoreductase	
		Co	aspartyl/glutamyl-tRNA (Asn/Gln) amidotransferase subunit B	-	
		Cd	ferredoxin-NADP oxidoreductase	-	
Bryophyta	*Fontinalis antipyretica Polytrichastrum formosum Sphagnum palustre*	Cd	-	GSH	[73]
Marchantiophyta	*Conocephalum conicum*	Cd	-	GSH	
Lycopodiophyta	*Selaginella denticulata*	Cd	-	GSH	
Anthocerotophyta	*Phaeoceros laevis*	Cd	GSH	-	
Pteridophyta	*Azolla imbricata, Azolla pinnata*	Cd	photosynthetic rate, chlorophyll *a*, *b*	anthocyanins, carotenoids, chalcone synthase, dihydroflavonol reductase, SOD, CAT, POD, lipid peroxidation	[58,59]
	*Azolla caroliniana, Salvinia minima*	Mn	chlorophylls	lipid peroxidation	[60]
	*Azolla filiculoides*	Mn	-	metallothionein gene expression	[74]
Magnoliophyta -Monocots	*Eichhornia crassipes*	Pb	-	lipid peroxidation, SOD, CAT, POD, APX	[75]
	*Oryza sativa*	Pb	CAT	lipid peroxidation, SOD, APX, GR, α-tocopherol	[70]
	*Pogonatherum crinitum*	Pb	-	lipid peroxidation, SOD, POD	[76]
	*Triticum aestivum*	Ni	chlorophylls	lipid peroxidation, proline, SOD	[61]
	*Zea mays*	Cu	-	SOD, CAT, APX, MAP- kinase activity	[77]
Magnoliophyta - Dicots	*Arabidopsis thaliana*	Cd	SOD, CAT, GR	lipid peroxidation, APX	[66]
		Cu	auxin level, CAT, shoot biomass	SOD, POD, chlorosis, necrosis and violet colouring of leaves	[55,78]
		Pb	-	CAT, POD, GPX, GSH	[79]
	*Cicer arietinum*	Cr	chlorophylls	-	[62]
	*Echium vulgare*	Cd	chlorophylls, carotenoids, proline, anthocyanins, phytochelatins, tartrate and succinate acids	-	[64]
	*Lonicera japonica*	Cd	GSH, APX, GR	net photosynthesis, PSII quantum efficiency, photochemical quenching, chlorophylls, carotenoids, CAT, lipid peroxidation, phytochelatins	[67,68,69]
	*Mentha arvensis*	Pb, Cd	chlorophylls	SOD, CAT, POD	[63]
	*Miscanthus sacchariflorus*	Cd, As	lipid peroxidation, POD	net photosynthesis, chlorophylls, stomatal conductance	[80]
	*Silene vulgaris*	Ni	growth rate, root and shoot development	-	[51]
		Pb	chlorophyll *a*, chlorophyll *b*	lipid peroxidation, phenols	
	*Spirodela polyrrhiza*	Cd, As	-	chlorophylls, carotenoids, non-protein thiols, ascorbic acid, cysteine and protein contents, biomass	[81]

Abbreviations: APX—ascorbate peroxidase; CAT—catalase; GPX—glutathione peroxidase; GR—glutathione reductase; GSH—reduced glutathione; MAP—mitogen-activated protein kinase; POD—guaiacol peroxidase; SOD—superoxide dismutase.

**Table 2:** Hyperaccumulating criteria for metallic elements (mg kg^−1^ of leaf dry weight) according to van der Ent et al. [14], main families, the total number of genera and examples of representatives. Based on Rascio and Navarii-Izo [157]; Reeves et al. [15].

Element	Minimal Concentration in Leaves (mg kg^−1^ DW)	Main Families and Their Total Number (in Bracket)	Genera Number	Examples of Species
Arsenic	1000	Pteridaceae (1)	2	*Pteris vittata, Pityrogramma calomelanos*
Cadmium	100	Brassicaceae, Crassulaceae (6)	7	*Arabidopsis halleri, Thlaspi* (*Noccaea*) *praecox, Solanum nigrum, Viola boachanensis*
Copper	300	Asteraceae, Commelinaceae, Fabaceae, Lamiaceae, Linderniaceae, Malvaceae, Orobanchaceae, Polygonaceae (20)	43	*Aeolanthus biformifolius, Anisopappus chinensis, Commoelina communis, Haumaniastrum katangense*
Cobalt	300	Asteraceae, Lamiaceae, Linderniaceae, Orobanchaceae, Phyllanthaceae (18)	34	*Crotalaria cobalticola, Haumaniastrum katangense, H. robertii*
Manganese	10,000	Celastraceae, Myrtaceae, Proteaceae (16)	24	*Gossia bidwillii, Virotia neurophylla*
Nickel	1000	Asteraceae, Brassicaceae, Buxaceae, Cunoniaceae, Phyllanthaceae, Salicaceae, Violaceae (52)	130	*Alyssum bertolonii, A. murale, A. lesbiacum, Berkhleya coddii, Phyllanthus cataractarum, Sebertia accuminata, Noccaea goesingense*
Lead	1000	Brassicaceae, Caryophyllaceae (6)	8	*Armeria maritima ssp. halleri, Minuartia verna, Noccaea rotondifolia subsp. cepaeifolia*
Selenium	100	Brassicaceae, Fabaceae (7)	15	*Astragalus bisulcatus, Hesperis persica, Stanleya pinnata*
Thallium	100	Brassicaceae (1)	2	*Biscutella laevigata, Iberis intermedia*
Zinc	3000	Brassicaceae, Crassulaceae (9)	12	*Anthyllis vulneraria, Arabidopsis halleri, Noccaea caerulescens, Sedum alfredii*
